# Capsular extension at ultrasound is associated with lateral lymph node metastasis in patients with papillary thyroid carcinoma: a retrospective study

**DOI:** 10.1186/s12885-021-08875-5

**Published:** 2021-11-20

**Authors:** Lei Ye, Lei Hu, Weiyong Liu, Yuanyuan Luo, Zhe Li, Zuopeng Ding, Chunmei Hu, Lin Wang, Yajuan Zhu, Le Liu, Xiaopeng Ma, Yuan Kong, Liangliang Huang

**Affiliations:** 1 Department of Ultrasound, Division of Life Science and Medicine, the First Affiliated Hospital of USTC, University of Science and Technology of China, No. 1, Tianehu Road, Hefei, 230036 Anhui China; 2 Department of Surgery, Division of Life Science and Medicine, the First Affiliated Hospital of USTC, University of Science and Technology of China, Hefei, 230036 Anhui China; 3 Department of Laboratory, Division of Life Science and Medicine, the First Affiliated Hospital of USTC, University of Science and Technology of China, Hefei, 230036 Anhui China; 4 Department of Pathology, Division of Life Science and Medicine, The First Affiliated Hospital of USTC, University of Science and Technology of China, Hefei, 230036 Anhui China

**Keywords:** Ultrasonography, Thyroid cancer, papillary, Metastasis, Lymph nodes

## Abstract

**Background:**

In patients with papillary thyroid cancer (PTC), cervical lymph node metastasis (LNM) must be carefully assessed to determine the extent of lymph node dissection required and patient prognosis. Few studies attempted to determine whether the ultrasound (US) appearance of the primary thyroid tumor could be used to predict cervical lymph node involvement. This study aimed to identify the US features of the tumor that could predict cervical LNM in patients with PTC.

**Methods:**

This was a retrospective study of patients with pathologically confirmed PTC. We evaluated the following US characteristics: lobe, isthmus, and tumor size; tumor position; parenchymal echogenicity; the number of lesions (i.e., tumor multifocality); parenchymal and lesional vascularity; tumor margins and shape; calcifications; capsular extension; tumor consistency; and the lymph nodes along the carotid vessels. The patients were grouped as no LNM (NLNM), central LNM (CLNM) alone, and lateral LNM (LLNM) with/without CLNM, according to the postoperative pathological examination.

**Results:**

Totally, 247 patients, there were 67 men and 180 women. Tumor size of > 10 mm was significantly more common in the CLNM (70.2%) and LLNM groups (89.6%) than in the NLNM group (45.4%). At US, capsular extension > 50% was most common in the LLNM group (35.4%). The multivariable analysis revealed that age (OR = 0.203, 95%CI: 0.095–0.431, *P* < 0.001) and tumor size (OR = 2.657, 95%CI: 1.144–6.168, *P* = 0.023) were independently associated with CLNM compared with NLNM. In addition, age (OR = 0.277, 95%CI: 0.127–0.603, *P* = 0.001), tumor size (OR = 6.069, 95%CI: 2.075–17.75, P = 0.001), and capsular extension (OR = 2.09, 95%CI: 1.326–3.294, P = 0.001) were independently associated with LLNM compared with NLNM.

**Conclusion:**

Percentage of capsular extension at ultrasound is associated with LLNM. US-guided puncture cytology and eluent thyroglobulin examination could be performed as appropriate to minimize the missed diagnosis of LNM.

## Background

Papillary thyroid cancer (PTC) is a carcinoma that arises from the thyroid follicular cells, is classified as differentiated thyroid cancer, and accounts for about 80% of all thyroid cancers [1]. PTC may be sporadic or genetic and can be subtyped as well-differentiated or poorly differentiated, with each subtype encompassing multiple variants, some of which are more aggressive than others [1]. In the United States, the incidence of PTC increased from 4.56 per 100,000 person-years in 1974–1977 to 14.42 per 100,000 person-years in 2010–2013 [2]. The mortality rate of PTC is low, but positive lymph nodes are associated with lower survival [3, 4].

Although PTC is an indolent cancer [3, 4], it is frequently associated with cervical lymph node metastasis (LNM) [3, 5]. The incidence of central compartment LNM in patients with PTC can be as high as 80% [6]. In PTC, LNM occurs in a stepwise fashion. Cells from the primary tumor in the thyroid gland first spread to the central and ipsilateral lateral lymph node compartments via lymphatic drainage and then to the mediastinal and contralateral lateral lymph node compartments [7–9]. This sequential pattern of LNM implies that patients with lateral LNM (LLNM) will typically also have central LNM (CLNM). Nevertheless, discontinuous lymphatic spread occasionally occurs, leading to “skip metastasis,” i.e., LLNM without CLNM [7–9]. Of course, the presence of LNM influences the prognosis and is associated with local tumor recurrence and poor patient survival [10]. Thus, in patients with PTC, cervical lymph node involvement must be carefully assessed to determine the extent of lymph node dissection required and patient prognosis.

Ultrasound (US) is the investigation of choice for both the pre- and postoperative surveillance in patients with PTC [11–13]. Grayscale US images can reveal highly specific features of the thyroid mass, such as microcalcifications and capsular extension, that are often impossible to detect using computed tomography (CT) or magnetic resonance imaging (MRI) [14–16]. In addition, compared with CT and MRI, US is inexpensive and fast, with no contraindications. Hence, it is beneficial to determine the significant ultrasound features that might indicate the presence of LNMs to help determine whether to perform central or lateral lymph node dissection.

Although the usefulness of the US to detect cervical LNM in PTC patients has been extensively investigated, few studies attempted to determine whether the US appearance of the primary thyroid tumor could be used to predict cervical lymph node involvement. Therefore, the present study aimed to identify the US features of the primary thyroid tumor used to predict cervical lymph node involvement in patients with PTC.

## Methods

### Study design and population

This retrospective study included patients with pathologically confirmed PTC who underwent preoperative thyroid US and surgical resection between April and September 2017 at the Anhui Province Hospital, The First Affiliated Hospital of the University of Science and Technology of China (Anhui Province, China). The study was approved by the Ethics Committee of the Anhui Province Hospital. Informed consent was waived due to the retrospective nature of this study.

The inclusion criteria were: 1) pathologically confirmed PTC; 2) underwent preoperative thyroid US; 3) underwent surgical resection; and 4) underwent lymph node dissection. The exclusion criteria were: 1) incomplete clinical data; or 2) US images were unclear or complete enough for analysis.

### Ultrasound

All US examinations were performed using an iU22 device (Philips Medical Systems, Best, The Netherlands) equipped with an L12–5 linear transducer. The transducer was placed transversely over the midline of the body to evaluate the position of the thyroid gland relative to the midline. Each thyroid lobe was imaged in the longitudinal and transverse axes. We evaluated the following characteristics on the US: lobe, isthmus, and tumor size; tumor position; parenchymal echogenicity; the number of lesions (i.e., tumor multifocality); parenchymal and lesional vascularity; tumor margins and shape; calcifications; capsular extension; tumor consistency; and the lymph nodes along the carotid vessels. The presence or absence of chronic lymphocytic thyroiditis was also assessed in the US and confirmed on pathological examination. The tumor shape was based on the ratio of the anteroposterior diameter to the transverse diameter. Microcalcifications were defined as multiple bright punctate echoes, each measuring < 1 mm, with or without acoustic shadows. The capsular extension was defined as the percentage of the perimeter of the thyroid nodule that was in contact with the thyroid capsule. Tumor echogenicity was described relative to the echogenicity of the surrounding muscle. The US examinations were performed by two physicians with more than 5 years of professional experience.

### Surgery

The interval between ultrasound and surgery was ≤3 days. All surgical procedures were performed by an experienced team of surgeons at the First Affiliated Hospital of the University of Science and Technology of China. As this is a retrospective study of consecutive patients, the patients were operated on by the surgeons operating thyroid cancer at our hospitals during the study period. The team included three associate chief physicians, nine attending physicians, and one resident physician (who was mainly an assistant). Unilateral thyroid lobectomy or isthmusectomy was performed in patients with unilateral cN0 PTC, while bilateral thyroid lobectomy or isthmusectomy was performed in patients with bilateral cN0 PTC. In addition, all patients underwent modified radical central and lateral lymph node dissection. Central lymph node dissection included the dissection of the pretracheal, prelaryngeal, and ipsilateral paratracheal lymph nodes. Lateral lymph node dissection involved the dissection of the lymph nodes in groups II to V while sparing the internal jugular vein, spinal accessory nerve, and sternocleidomastoid muscle. Lymph node metastases (macrometastases and micrometastases) and chronic lymphocytic thyroiditis were confirmed using the postoperative pathological examination. Lateral neck dissection was performed in the following cases: 1) US indicated suspicious lymph node metastasis; even though biopsy results were negative, dissection was performed considering the US results; 2) during the operation, it was found that the tumor was large and the lymph nodes in the central region were involved; or 3) the tumor was large and close to the superior lymph nodes, and lymph node metastasis in regions II and III are frequent, and dissection was performed for prevention. Pathological results were determined by two pathologists with more than 5 years of experience. The pathologists were blind to the US data.

### Grouping

The patients were divided into three groups based on their lymph node status: (i) no LNM, NLNM group; (ii) CLNM alone without LLNM, CLNM group; and (iii) LLNM with or without CLNM, LLNM group. CLNM was defined as metastasis to the group VI lymph nodes (i.e., the central lymph nodes). LLNM was defined as the involvement of the lymph nodes in groups II to V (i.e., the lateral lymph nodes) [17].

### Observational indexes

**Fig. 1:**
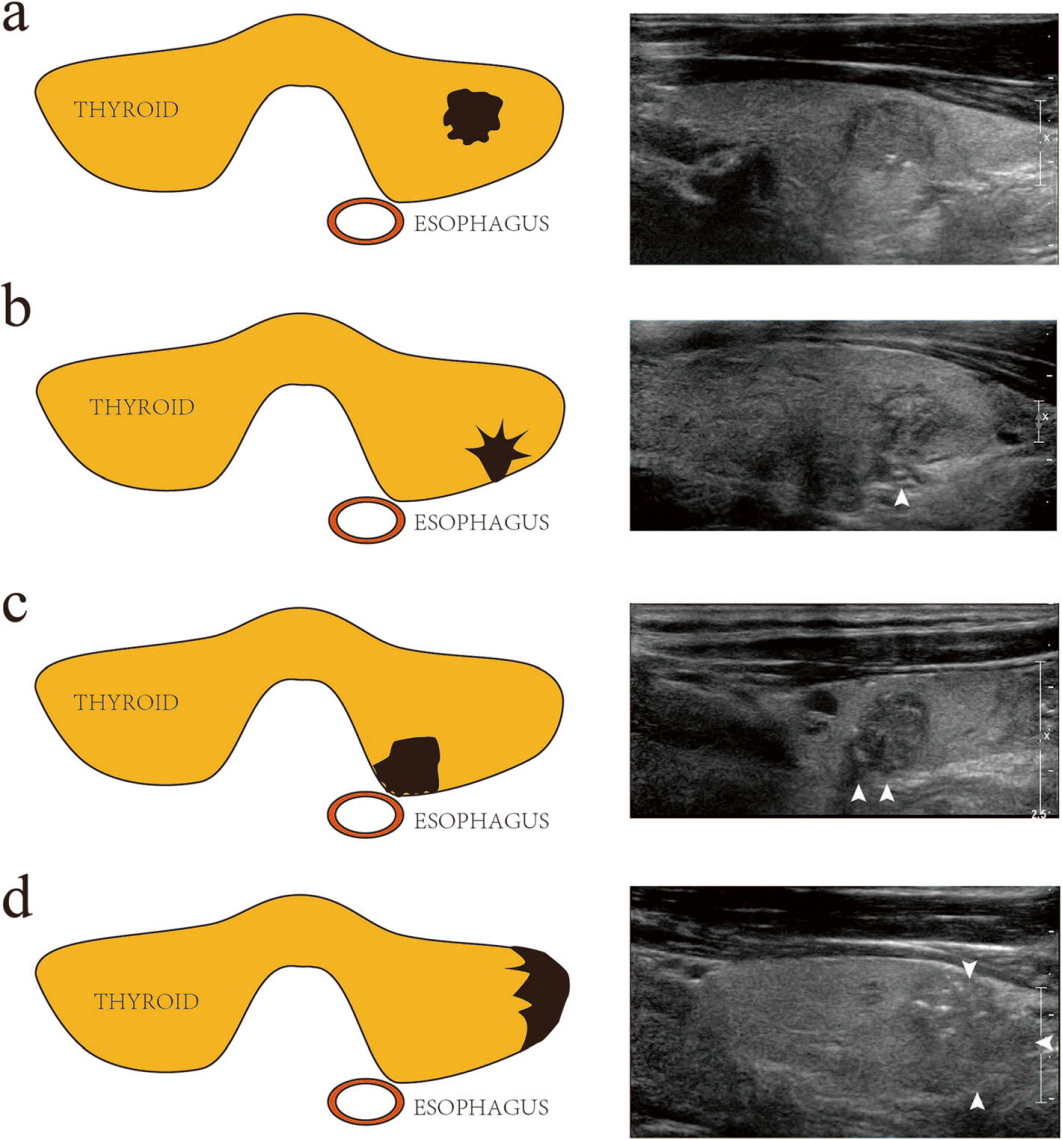
Classification of capsular extension in papillary thyroid cancer: no contact (**a**), < 25% contact (**b**), 25–50% contact (**c**), and > 50% contact (**d**)

The demographic information like age and gender and all clinical, imaging, and pathological features were collected from the clinical records. The following factors were evaluated as potential predictors of LNM in patients with PTC: maximum tumor dimension (≤10 mm vs. > 10 mm) [18], age (< 45 years vs. ≥45 years) [19], tumor position (upper, middle, or lower third of the thyroid, or the thyroid isthmus), tumor multifocality (single lesion vs. multiple lesions), tumor margins (regular vs. irregular), tumor shape (round-to-ovoid vs. taller-than-wide), calcification (absent, microcalcification, or other types), capsular extension (0, < 25, 25–50%, and > 50%; Fig. 1), echogenicity (isoechoic, hypoechoic, or markedly hypoechoic), tumor consistency (solid vs. mixed solid-cystic), and chronic lymphocytic thyroiditis (absent vs. present). A taller-than-wide shape was identified when the ratio of the anteroposterior diameter to the transverse diameter was ≥1. Data were double entered to ensure exactness and minimize bias.

### Statistical analysis

Statistical analyses were performed using SAS 9.1 for Windows (SAS Institute, Cary, NY, USA). Continuous variables were presented as means ± standard deviations (SD) or as medians and interquartile ranges (IQR), according to their distribution, as determined by the Kolmogorov-Smirnov test. The continuous variables were compared by ANOVA, with the least significant difference (LSD) test and the Bonferroni method for pairwise comparison. Categorical variables were reported as frequencies and percentages and were compared using the chi-square or Fisher’s exact test. The incidence of individual US features was analyzed using the Cochran-Mantel-Haenszel chi-square test. Logistic regression analysis was performed to estimate the association of certain parameters with LNM, and the results were presented as odds ratios (OR) with a 95% confidence interval (CI). Differences were considered statistically significant at *P* < 0.05.

## Results

### General characteristics

A total of 372 patients underwent preoperative thyroid US for the assessment of a thyroid nodule. Postoperative pathological examination confirmed PTC in 247 of these patients, who were included in the study. Among the 247 patients, there were 67 men and 180 women. The mean age was 44.2 ± 11.6 years (range, 12–76 years). The patients were grouped as NLNM group, 152 patients; CLNM group, 47 patients; and LLNM, 48 patients (LLNM+CLNM, 31 patients; LLNM alone, 17 patients). The general characteristics of the patients are shown in Table 1. Sex distribution did not differ among the NLNM (37 men, 115 women), CLNM (14 men, 33 women), and LLNM groups (16 men, 32 women; *P* = 0.427). The patients in the CLNM (mean age, 39.7 ± 10.9 years) and LLNM groups (40.1 ± 10.8 years) were significantly younger than those in the NLNM group (46.9 ± 11.3 years) (*P* < 0.001).

**Table 1:** Clinicopathological characteristics of the patients with papillary thyroid carcinoma (*n* = 247)

Characteristics	NLNM (*n* = 152)	CLNM (*n* = 47)	LLNM (*n* = 48)	*P*
Age (years), mean ± SD	46.9 ± 11.3	39.7 ± 10.9	40.1 ± 10.8	< 0.001
< 45	56 (36.8)	34 (72.3)	31 (64.6)	< 0.001
≥ 45	96 (63.2)	13 (27.7)	17 (35.4)	
Sex (male), n (%)	37 (24.3)	14 (29.8)	16 (23.3)	0.427
Hashimoto’s thyroiditis, n (%)	35 (23.0)	15 (31.9)	13 (29.2)	0.446
Tumor size (mm), median (IQR)	9.4 (7,14)	15 (10,21)	18 (14,23.75)	< 0.001
≤ 10	83 (54.6)	14 (29.8)	5 (10.4)	< 0.001
> 10	69 (45.4)	33 (70.2)	43 (89.6)	
Tumor location, n (%)				
Upper third	29 (19.1)	8 (17.0)	9 (18.7)	0.945^f^
Middle third	86 (56.6)	24 (51.1)	26 (54.2)	
Lower third	35 (23.0)	14 (29.8)	12 (25.0)	
Isthmus	2 (1.3)	1 (2.1)	1 (2.1)	
Pathological multifocality, n (%)				
Single	129 (84.9)	35 (74.5)	37 (77.1)	0.643
Multiple	23 (15.1)	12 (25.5)	11 (22.9)	
Pathological type, n (%)				
Papillary microcarcinoma	64 (42.1)	18 (38.3)	24 (50.0)	0.345^f^
Papillary carcinoma	86 (56.6)	28 (59.6)	24 (50.0)	
Medullary carcinoma	0	1 (2.1)	0	
Follicular adenocarcinoma	2 (1.3)	0	0	

^f^ Fisher’s exact test

*NLNM* no lymph node metastasis, *CLNM* central lymph node metastasis, *LLNM* lateral lymph node metastasis, *SD* standard deviation; IQR: interquartile range

### Factors associated with LNM

The frequencies of tumor location, multifocality, and chronic lymphocytic thyroiditis did not differ among the three groups (all *P* > 0.05). Tumor size of > 10 mm was significantly more common in the CLNM (70.2%) and LLNM groups (89.6%) than in the NLNM group (45.4%) (P < 0.001). Most tumors had irregular margins, microcalcifications, hypoechoic appearance, and solid consistency, and the frequency of these four characteristics did not differ among the three groups (*P* = 0.825, *P* = 0.240, *P* = 0.943, and *P* = 0.640, respectively). A round-to-ovoid shape was significantly more common in the LLNM group (81.3%) than in the NLNM (61.2%) and CLNM groups (76.6%) (*P* = 0.013). Capsular extension > 50% was most common in the LLNM group (35.4%) (*P* < 0.001) (Table 2).

**Table 2:** Patients and ultrasound findings of papillary thyroid carcinoma (*n* = 247)

Characteristics, n (%)	NLNM (*n* = 152)	CLNM (*n* = 47)	LLNM (*n* = 48)	*P*
Shape				
Regular	45 (29.6)	9 (19.2)	12 (25.0)	0.351
Irregular	107 (70.4)	38 (80.9)	36 (75.0)	
Margin				
Smooth	9 (5.8)	1 (2.1)	3 (6.3)	0.825^f^
Non-smooth	143 (94.2)	46 (97.9)	45 (93.8)	
Aspect ratio				
< 1	93 (61.2)	36 (76.6)	39 (81.3)	0.013
> 1	59 (38.8)	11 (23.4)	9 (18.7)	
Calcification				
Absent	35 (22.9)	8 (17.0)	6 (12.5)	0.240^f^
Macrocalcification	102 (68.0)	37 (78.7)	35 (72.9)	
Microcalcification	14 (9.1)	2 (4.3)	7 (14.6)	
Extension toward the capsule				
0	42 (27.6)	9 (19.2)	3 (6.3)	< 0.001^f^
< 25%	43 (28.3)	13 (27.7)	7 (14.6)	
25–50%	55 (36.2)	16 (34.0)	21 (43.8)	
> 50%	12 (7.9)	9 (19.1)	17 (35.4)	
Echo				
Isoechoic	2 (1.3)	0 (0)	0 (0)	0.943^f^
Hypoechoic	142 (93.4)	45 (95.7)	42 (87.5)	
Markedly hypoechoic	8 (5.3)	2 (4.3)	6 (12.5)	
Internal structure				
Solid	150 (98.7)	46 (97.9)	48 (100.0)	0.640^f^
Mixed solid-cystic	2 (1.3)	1 (2.1)	0 (0)	

^f^ Fisher’s exact test

*NLNM* no lymph node metastasis, *CLNM* central lymph node metastasis, *LLNM* lateral lymph node metastasis

### Multivariable logistic regression analysis

A multivariable logistic regression analysis of the factors that significantly differed between the three groups was performed (Table 3). The analysis revealed that age (OR = 0.203, 95%CI: 0.095–0.431, P < 0.001) and tumor size (OR = 2.657, 95%CI: 1.144–6.168, *P* = 0.023) were independently associated with CLNM compared with NLNM. In addition, age (OR = 0.277, 95%CI: 0.127–0.603, *P* = 0.001), tumor size (OR = 6.069, 95%CI: 2.075–17.75, P = 0.001), and percentage of capsular extension (OR = 2.09, 95%CI: 1.326–3.294, P = 0.001) were independently associated with LLNM compared with NLNM.

**Table 3:** Multivariable logistic regression analysis for the presence of CLNM or LLNM compared with NLNM (*n* = 247)

Characteristics	OR	95%CI	*P*
CLNM			
Age (year)	0.203	0.095–0.431	< 0.001
Tumor size (mm)	2.657	1.144–6.168	0.023
Extension toward the capsule	1.197	0.809–1.772	0.368
Aspect ratio > 1	0.878	0.374–2.064	0.766
LLNM			
Age (year)	0.277	0.127–0.603	0.001
Tumor size (mm)	6.069	2.075–17.75	0.001
Extension toward the capsule	2.09	1.326–3.294	0.001
Aspect ratio > 1	1.006	0.394–2.573	0.989

*OR* odds ratio, *CI* confidence interval, *NLNM* no lymph node metastasis, *CLNM* central lymph node metastasis, *LLNM* lateral lymph node metastasis

## Discussion

Given that few studies attempted to determine whether the ultrasound appearance of the primary thyroid tumor could be used to predict cervical LNM, this study aimed to identify the ultrasound features of the primary thyroid tumor that could be associated with cervical LNM in PTC. The results suggest that age and tumor size were independently associated with CLNM in patients with PTC. Age, tumor size, and percentage of capsular extension at ultrasound were independently associated with LLNM.

This study revealed that among patients with suspected PTC, age < 45 years, tumor size > 10 mm, and capsular extension > 50% were independently associated with LNM. The overall incidence of cervical LNM in the present study was approximately 38.5% (95/247), consistent with previous reports [6]. The incidence of skip metastasis (i.e., LLNM without CLNM) was 6.9% (17/247). Women accounted for more than 70% of the patients in each group, and the sex distribution was similar among the three groups. Although PTC incidence is higher among women, men require specialized thyroid checkups to enable the early detection of thyroid tumors [20]. Younger age (< 45 years) has been associated with an increased risk of CLNM [20]. In a previous study, the univariable analysis showed that LNM is more likely among men, patients younger than 45 years, patients with multifocal tumors, and patients with local infiltration [21]. Consistent with this, the present study showed that patients were more likely to be younger than 45 years old in the CLNM (72.3%) and LLNM groups (64.6%) than in the NLNM group (36.8%).

Tumor shape and capsular extension at ultrasound were related to cervical LNM, but tumor margins, calcification, echogenicity, and consistency were not. Previous studies reported that a taller-than-wide shape is a useful predictor of thyroid malignancy [22–24]. The dense fibrosis in a PTC may lead to decreased compressibility and a taller-than-wide shape [23]. In the present study, a taller-than-wide shape was significantly more common in the NLNM group (38.8%) than in the CLNM (23.4%) or LLNM group (18.7%).

Capsular extension, specifically the degree of capsular extension and capsular disruption, can predict extrathyroidal extension and invasive thyroid cancer [25]. Skip metastases are more common in patients with PTC and primary tumor capsular invasion than patients without capsular invasion [26]. In the present study, capsular extension > 50% was the most common in the LLNM group (35.4%). Irregular tumor margins are a sign of malignancy. In this study, the incidence of non-smooth margins was more than 90% in all three groups. This high incidence might be attributable to the use of the high-resolution US and strict observation standards. Microcalcification was frequently observed in the present study, but its incidence did not significantly differ among the three groups. Only a few studies included capsular extension as one of the US features [5, 14, 26, 27]. The present study suggests that capsular invasion is significantly associated with LLNM. In other words, when a capsular extension is found by US, the patient should be considered at a relatively high risk of LLNM, and the surgeons might consider performing lateral lymph node dissection.

Tumor size is associated with the extent of cancer cell proliferation, and a faster rate of cell proliferation correlates with a higher risk of CLNM [28]. Accordingly, tumor size is a strong predictor of microscopic CLNM and LLNM in N0 PTC patients [29]. In the present study, tumor size was largest in the LLNM group (*P* < 0.001). A tumor size > 10 mm was more frequent in the CLNM (70.2%) and LLNM groups (89.6%) than in the NLNM group (45.4%).

A recent study revealed that Delphian lymph node (DLN) metastasis in PTC patients was related to tumor location in the isthmus or upper third of the thyroid [28]. Azizi et al. [30] found that thyroid nodules in the isthmus were more likely to be malignant than nodules elsewhere in the gland. In the present study, 55.1% (136/247) of the patients had tumors in the middle third of the thyroid. Only four patients had tumors located in the isthmus. Tumor location did not significantly differ among the three groups.

Most patients with PTC (18–87%) have multifocal tumors. Some studies found that multifocality is associated with a high risk of LNM among patients with thyroid microcarcinomas, but other studies reported that the risk of LNM does not differ between those with unifocal and multifocal PTC [21, 31]. In the present study, the incidence of multifocality did not differ among the three groups.

Hashimoto’s thyroiditis is the most common form of autoimmune thyroid disease, with an incidence rate of about 2% in the general population. Some investigators reported that Hashimoto’s thyroiditis is a risk factor for PTC, while others found no correlation between the two [32]. A study revealed that the incidence of Hashimoto’s thyroiditis among PTC patients ranges from 9 to 58% [27]. In the present study, the incidence of Hashimoto’s thyroiditis did not significantly differ among the three groups.

Partially cystic and iso- or hyperechoic nodules are generally benign, with a low malignancy risk. In the present study, most of the tumors were hypoechoic, and the incidence of hypoechogenicity did not differ among the three groups. PTC is almost always solid, and cystic changes in PTC are rare. Some studies showed that mixed echogenicity (cystic component > 50%), accompanied by a honeycomb appearance, is an indication of benignity [22].

There are several potential limitations to this study. First, this is a retrospective study, and further prospective study is necessary to confirm the results. Second, we did not compare the US features of lymph nodes with and without metastasis. Third, we did not examine the association between capsular extension at ultrasound and the actual capsular contact at the histological level. Fourth, the signal intensity-related parameters showed inter-individual differences and were affected by certain conditions. Fifth, the sub-types of PTC were not recorded. Sixth, the BRAF V600E mutation was not examined. Finally, only patients with PTC were included in this study and not patients with medullary or undifferentiated carcinomas. This study, therefore, does not represent all the pathological subtypes of thyroid carcinoma.

## Conclusion

Age and the size of the thyroid cancer lesion might be related to CLNM and LLNM. In addition, a capsular extension of nodules and thyroid, which is examined in only a few studies, is associated with LLNM. When suspicious lymph nodes are found preoperatively, US-guided puncture cytology and eluent thyroglobulin examination could be performed to minimize the missed diagnosis of LNM.

## Data Availability

The datasets used and/or analyzed during the current study are available from the corresponding author on reasonable request.

## Abbreviations

PTC: Papillary thyroid cancer
NLNM: no lymph node metastasis
CLNM: central LNM
LLNM: lateral LNM
US: Ultrasound
CT: computed tomography
MRI: magnetic resonance imaging
DLN: Delphian lymph node
