# Correction to: Capsular extension at ultrasound is associated with lateral lymph node metastasis in patients with papillary thyroid carcinoma: a retrospective study

**DOI:** 10.1186/s12885-022-09303-y

**Published:** 2022-02-21

**Authors:** Lei Ye, Lei Hu, Weiyong Liu, Yuanyuan Luo, Zhe Li, Zuopeng Ding, Chunmei Hu, Lin Wang, Yajuan Zhu, Le Liu, Xiaopeng Ma, Yuan Kong, Liangliang Huang

**Affiliations:** 1 Department of Ultrasound, the First Affiliated Hospital of USTC, Division of Life Science and Medicine, University of Science and Technology of China, No. 1, Tianehu Road, Hefei, 230036 Anhui China; 2 Department of Laboratory, Division of Life Science and Medicine, the First Affiliated Hospital of USTC, University of Science and Technology of China, Hefei, 230036 Anhui China; 3 Department of Surgery, Division of Life Science and Medicine, the First Affiliated Hospital of USTC, University of Science and Technology of China, Hefei, 230036 Anhui China; 4 Department of Pathology, Division of Life Science and Medicine, The First Affiliated Hospital of USTC, University of Science and Technology of China, Hefei, 230036 Anhui China

**Correction to: BMC Cancer 21 (2021)**

**https://doi.org/10.1186/s12885-021-08875-5**

Following publication of the original article [1], the authors identified an error in affiliation 1.

The correct affiliation 1 is:

Department of Ultrasound, the First Affiliated Hospital of USTC, Division of Life Science and Medicine, University of Science and Technology of China, No. 1, Tianehu Road, Hefei 230036, Anhui, China
